# Supplementary material for: The efficacy of conditioned medium released by tonsil-derived mesenchymal stem cells in a chronic murine colitis model
Source: PLoS One. 2019 Dec 2;14(12):e0225739. doi: 10.1371/journal.pone.0225739 (PMC6886802; doi:10.1371/journal.pone.0225739)
Supplement: S1 Table — (DOCX) [file pone.0225739.s001.docx]

**S1 Table. Disease Activity Index (DAI) Scoring System**

| **Score** | **Weight loss** | **Stool consistency** | **Occult/gross rectal bleeding** |
| --- | --- | --- | --- |
| 0 | None | Normal | Normal |
| 1 | 1-5% | - | - |
| 2 | 5-10% | Loose stool | Hemoccult |
| 3 | 10-20% | - | - |
| 4 | > 20% | Diarrhea | Gross bleeding |
