# Supplementary material for: The efficacy of conditioned medium released by tonsil-derived mesenchymal stem cells in a chronic murine colitis model
Source: PLoS One. 2019 Dec 2;14(12):e0225739. doi: 10.1371/journal.pone.0225739 (PMC6886802; doi:10.1371/journal.pone.0225739)
Supplement: S2 Table — (DOCX) [file pone.0225739.s002.docx]

**S2 Table. Histologic Colitis Scoring System**

| **Feature score** | **Score** | **Description** |
| --- | --- | --- |
| Inflammation severity | 0 | None |
|  | 1 | Mild |
|  | 2 | Moderate |
|  | 3 | Severe |
| Inflammation extent | 0 | None |
|  | 1 | Mucosa |
|  | 2 | Submucosa |
|  | 3 | Transmural |
| Crypt damage | 0 | None |
|  | 1 | Basal 1/3 damage |
|  | 2 | Basal 2/3 damage |
|  | 3 | Crypt lost ; surface epithelium present |
|  | 4 | Crypt and surface epithelium lost |
| Percent involvement | 0 | 0% |
|  | 1 | 1-25% |
|  | 2 | 26-50% |
|  | 3 | 51-75% |
|  | 4 | 76-100% |
