# Supplementary material for: The efficacy of conditioned medium released by tonsil-derived mesenchymal stem cells in a chronic murine colitis model
Source: PLoS One. 2019 Dec 2;14(12):e0225739. doi: 10.1371/journal.pone.0225739 (PMC6886802; doi:10.1371/journal.pone.0225739)
Supplement: S3 Table — (DOCX) [file pone.0225739.s003.docx]

**S3 Table. Disease activity index (DAI), weight change, and colon length at the 30th day of experiment**

|  | DAI | Weight change (%) | Colon length (cm) |
| --- | --- | --- | --- |
| Normal | 0 | 17.55 ± 4.19 | 93.96 ± 55.57.85 |
| Colitis | 4.44 ± 3.91 | -2.69 ± 19.79 | 72.21 ± 6.55 |
| TMSC | 1.80 ± 0.54 | 9.25 ± 6.53 | 80.68 ± 5.87 |
| TMSC-CM | 1.54 ± 0.69 | 11.12 ± 12.12 | 80.5 ± 7.05 |
| TMSC-CM-conc | 1.28 ± 0.67 | 13.57 ± 7.20 | 81.8 ± 5.57 |
| *P*-value (ANOVA) | 0.0022 | 0.0183 | 0.0014 |
