# Supplementary material for: The efficacy of conditioned medium released by tonsil-derived mesenchymal stem cells in a chronic murine colitis model
Source: PLoS One. 2019 Dec 2;14(12):e0225739. doi: 10.1371/journal.pone.0225739 (PMC6886802; doi:10.1371/journal.pone.0225739)
Supplement: S4 Table — (DOCX) [file pone.0225739.s004.docx]

**S4 Table. Histologic colitis scoring at the 30th day of experiment**

|  | Histologic scoring |
| --- | --- |
| Normal | 0 |
| Colitis | 11.39 ± 2.81 |
| TMSC | 11.83 ± 2.09 |
| TMSC-CM | 11.11 ± 2.55 |
| TMSC-CM-conc | 11.93 ± 1.98 |
| *P*-value (ANOVA) | 0.2933 |
