# Supplementary material for: The efficacy of conditioned medium released by tonsil-derived mesenchymal stem cells in a chronic murine colitis model
Source: PLoS One. 2019 Dec 2;14(12):e0225739. doi: 10.1371/journal.pone.0225739 (PMC6886802; doi:10.1371/journal.pone.0225739)
Supplement: S5 Table — (DOCX) [file pone.0225739.s005.docx]

**S5 Table. Level of cytokine at the 30th day of experiment**

|  | IL-1β | IL-6 | IL-17 | TNFα | IL-10 |
| --- | --- | --- | --- | --- | --- |
| Normal | 1.15 ± 0.71 | 1.09 ± 0.46 | 1.63 ± 2.02 | 1.32 ± 0.92 | 2.29 ± 5.00 |
| Colitis | 68.36 ± 55.53 | 32.12 ± 34.41 | 42.19 ± 44.95 | 9.58 ± 7.91 | 23.07 ± 50.98 |
| TMSC | 21.66 ± 31.37 | 5.42 ± 3.10 | 18.64 ± 32.91 | 5.20 ± 3.68 | 15.10 ± 22.10 |
| TMSC-CM | 30.27 ± 35.89 | 14.24 ± 17.06 | 14.34 ± 12.54 | 5.47 ± 4.21 | 7.33 ± 4.19 |
| TMSC-CM-conc | 20.10 ± 18.56 | 4.95 ± 3.01 | 9.8 ± 6.88 | 5.54 ± 3.25 | 6.41 ± 2.52 |
| *P*-value  (ANOVA) | 0.0188 | 0.0103 | 0.0456 | 0.1611 | 0.4506 |

IL, interleukin; TNF, tumor necrosis factor
